# Tangduqing Granules Attenuate Insulin Resistance and Abnormal Lipid Metabolism through the Coordinated Regulation of PPAR*γ* and DGAT2 in Type 2 Diabetic Rats

**DOI:** 10.1155/2019/7403978

**Published:** 2019-03-25

**Authors:** Qinghua Zhang, Yingying Huang, Xiaojin Li, Hongyi Liu, Baichuan He, Bin Wang, Yuntao Ma, Xiang Zhou, Yaqin Liu, Shentao Wu

**Affiliations:** ^1^First Teaching Hospital of Tianjin University of Traditional Chinese Medicine, Tianjin, China; ^2^Tianjin University of Traditional Chinese Medicine, Tianjin, China; ^3^Yunnan Provincial Hospital of Traditional Chinese Medicine, Yunnan, China; ^4^Tianjin Nankai hospital, Tianjin, China

## Abstract

Insulin resistance (IR) is a vital hallmark of type 2 diabetes mellitus, which is characterized by an impaired ability of insulin to promote glucose uptake and utilization. Lipid deposition is closely associated with impaired insulin sensitivity. PPAR*γ* plays an important role in glucose homeostasis, adipocyte differentiation, and insulin sensitivity. Likewise, DGAT2 also exerts a crucial role in integrating carbohydrate and lipid metabolism in the liver. The present study is aimed at evaluating a Chinese medicinal formula, Tangduqing granules (TDQ), with multifaceted actions against lipid and glucose metabolism disorder and IR of type 2 diabetes. An animal model of type 2 diabetes was developed by high-fat diet feeding plus low-dose streptozotocin injection. After oral administration of TDQ for 5 weeks, the effects on glucose and lipid metabolism and the underlying mechanism were evaluated by biochemical, histological, RT-PCR, and western blotting methods. The results showed that TDQ decreased fasting blood glucose, ameliorated glucose tolerance, and improved IR. Besides, TDQ regulated hyperlipidemia symptoms, decreased serum lipid levels and liver TG, and reduced hepatic steatosis in a type 2 diabetic rat model. Furthermore, TDQ reversed diabetes-induced decrease in the mRNA and protein expression of PPAR*γ* and elevation in the mRNA and protein levels of DGAT2 in the liver. In addition, we showed that interference of TDQ ameliorated palmitate-induced glucose and lipid metabolic abnormalities in HepG2 cells. TDQ are, therefore, a potential Chinese medicinal formula that relieves IR and lipid metabolism disorder might be through promotion of PPAR*γ* and decrease of DGAT2 expression.

## 1. Introduction

The global incidence of diabetes is expected to rise to 592 million by 2035, and type 2 diabetes mellitus will be more than 500 million, indicating that these pathologies will continue to impact human health [1]. IR is a vital factor in the pathogenesis and etiology of type 2 diabetes mellitus, which is characterized by an impaired ability of insulin to promote glucose uptake and utilization [2]. Moreover, lipid accumulation is closely associated with impaired insulin sensitivity. In terms of inhibition of hepatic glucose production and stimulation of glycogen synthesis, the steatotic liver is resistant to insulin [3–5]. As the center of glucose and lipid metabolism and the main target organ for insulin, the liver plays an important role in IR.

Extensive studies have demonstrated that peroxisome proliferator-activated receptor *γ* (PPAR*γ*) is a member of the nuclear hormone receptor family and plays a critical role in lipid and glucose metabolism, such as adipocyte differentiation, glucose homeostasis, and insulin sensitivity. Additionally, PPAR*γ* is also involved in inflammatory response, macrophage cholesterol metabolism, and energy metabolism [6, 7]. Accordingly, pharmacological activation of PPAR*γ* has emerged as an effective method for treating diabetes, atherosclerosis, and other metabolic diseases [8–10]. Thiazolidinedione (PPAR*γ* agonist) is used to improve circulating glucose levels and insulin resistance in diabetic patients, but it was pushed back to an optional second-tier drug for unwanted adverse effects [10]. Nevertheless, PPAR*γ* is still an important target for the treatment of hyperlipidemia, insulin resistance, and diabetes mellitus. Moreover, diacylglycerol acyltransferase 2 (DGAT2) also plays a crucial role in integrating carbohydrate, lipid metabolism, and IR in the liver [11–13]. However, suppression of DGAT2 pharmacologically in the liver has been proved to reverse hepatic steatosis, insulin resistance, and hypertriglyceridaemia [12].

The complicated pathophysiology of type 2 diabetes means that a treatment needs to involve multiple therapies to regulate glucose and lipid homeostasis, as well as IR. However, a multitherapy treatment may result in drug effectiveness decreasing or toxicity enhancing and lead to drug interactions or compromised patient compliance [14, 15]. Hence, the development of novel antidiabetic drugs is emerging, such as dual-action drugs [15]. However, traditional Chinese medicine formula shows a unique advantage in the complex diseases, because the formula with multicomponents may work on multitargets. Tangduqing granules (TDQ) were a Chinese medicinal formula prepared in a hospital, which is comprised of several Chinese herbs with complex components: Coptis chinensis Franch., *Rheum palmatum* L., Scutellaria baicalensis Georgi, *Paeonia lactiflora* Pall, and so on. Previous reports revealed that TDQ work effectively against lipid and glucose metabolism disorder in clinical setting [16–18]. However, the effect and mechanism of TDQ on IR remain elusive. The present study hypothesizes that TDQ, acting as a PPAR*γ* agonist and a DGAT2 inhibitor, not only alleviate glucose and lipid metabolism disorder but also ameliorate IR in type 2 diabetic rats.

## 2. Materials and Methods

### 2.1. Drugs

TDQ (Jin Z20160067) were prepared at the First Affiliated Hospital of Tianjin University of Traditional Chinese Medicine. It contained 119 g *Rheum palmatum L.* (Rhei Radix et Rhizoma, Dahuang), 178 g *Coptis chinensis Franch.* (Coptidis Rhizoma, Huanglian), 149 g *Curcuma longa L.* (Curcumae Longae Rhizoma, Jianghuang), 109 g *Cryptotympana pustulata Fabricius* (Cicadae Periostracum, Chantui), 71 g *Bombyx mori Linnaeus* (Bombyx Batryticatus, Jiangcan), 71 g *Citrus auranium L.* (Aurantii Fructus Immaturus, Zhishi), 89 g *Pinellia ternata (Thunb.) Makino* (Pinelliae Rhizoma Praeparatum Cum Alumine, Qingbanxia), 149 g *Paeonia lactiflora Pall.* (Paeoniae Radix Alba, Baishao), 119 g *Bupleurum chinense DC.* (Bupleuri Radix, Chaihu), 149 g *Scutellaria baicalensis Georgi* (Scutellariae Radix, Huangqin), 59 g *Zingiber officinale Roscoe* (Zingiberis Rhizoma, Ganjiang), and 119 g *Eupatorium fortunei Turcz.* (Eupatorii Herba, Peilan) in every 1000 g granules. All the herbs were crushed into fine powder, extracted with distilled water for 2 h thrice, mixed with filtrate, and then dried at 55 ± 5°C in a vacuum, concentrated to a relative density of 1.09~1.14. Then, we added ethanol to make 60% alcohol content, got the supernatant, and recovered the ethanol, concentrated to a relative density of 1.28~1.32. Next, the extractum was mixed with dextrin, granulated with ethanol, and dried to 1000 g, which is equivalent to 1.38 grams of crude medicine per gram. The quality control standard of TDQ is that the total amount of baicalin (molecular formula is C_21_H_18_O_12_) should not be lower than 5.0 mg analyzed by HPLC as a reference in 1 g granules. For an experimental study, the granules was dissolved with distilled water to the appropriate concentration for further application. Pioglitazone hydrochloride tablets were provided by Japan Takeda Pharmaceutical Industry Co. Ltd., grinded into powder before use, and dissolved with distilled water according to the experimental concentration.

### 2.2. Animals and Treatment

Wistar rats (certificate No. SCXK 2009-0015) were purchased from Huafukang Bioscience Co. Ltd. (Beijing, China). Animals were kept in an environmentally controlled breeding room (temperature: 23 ± 2°C, humidity: 55 ± 10%, 12 h dark/light cycles). Water and food were provided ad libitum. All experiments were carried out in strict accordance with the Guide for the Care and Use of Laboratory Animals, and the protocol was approved by the Animal Ethics Committee of Tianjin University of Traditional Chinese Medicine (No. TCM-2015-020-E03).

Male Wistar rats, weighing 180–220 g, after a 7-day acclimatization to laboratory conditions, were randomly divided into two groups: normal group (Nor, *n* = 10) and high-fat diet group (HFD, in which contained 60% fat, 20% protein, and 20% carbohydrate, *n* = 100), which lasted for 12 weeks. Then, the rats were fasted for 12 h, HFD rats were subjected to a single injection of 28 mg/kg streptozotocin (STZ, Sigma-Aldrich, St Louis, MO, USA) freshly dissolved in 0.1 mol/L sodium citrate buffer at pH 4.5, and normal rats were injected once with vehicle citrate buffer [19]. Three days after STZ injection, the value of fasting blood glucose (FBG) was determined using blood glucose test strips (ROCHEN, Germany) in the serum to be tested. The rats with FBG levels above 11.1 mmol/L were considered diabetic [20], and then, they were randomly divided into five groups (*n* = 14 per group): model group (Mod, ig. distilled water), TDQ low-dose group (TDQ-L, ig. 1.5 g/kg daily), TDQ middle-dose group (TDQ-M, ig. 3.0 g/kg daily), TDQ high-dose group (TDQ-H, ig. 6.0 g/kg daily), and pioglitazone group (PIO, ig. 3.0 mg/kg daily). Each drug was administrated for 5 weeks.

In the preparation of TDQ-containing serum, 40 Wistar rats, weighing 230–250 g (20 males and 20 females), were randomly arranged in 2 groups. The TDQ group was gavaged with TDQ in doses of 6 g/kg/d, while rats of the vehicle group were gavaged with volume-matched distilled water. TDQ were administrated for 4 days. On day 5, after 12 h of fasting, rats were subjected to the last administration, and 1 h postadministration, the blood was retrieved via aorta ventralis, with anesthesia by intraperitoneal injection of pentobarbital sodium at a dose of 50 mg/kg. Then, sera were collected by centrifugation of blood at 3000 r/min for 15 min at 4°C, inactivated at 56°C. Finally, all rat sera were sterilized by filtration through 0.22 *μ*m cellulose ester membranes and stored at -20°C until further use.

### 2.3. Cell Culture and Treatment

Human hepatoma HepG2 cells (Cell Resource Center, Shanghai Institute of Life Sciences, Chinese Academy of Sciences) were maintained in Dulbecco's modified Eagle's medium (DMEM, HyClone, USA) supplemented with 15% FBS, 100 U/mL penicillin, and streptomycin (HyClone, USA), at 37°C in an incubator containing 5% CO_2_. To establish a hepatocellular model of insulin resistance, HepG2 cells were treated with 0.5 mmol/L palmitate (PA, Sigma) for 24 h, as follows: cells were cultured in a 6-well plate, when reaching 80% confluence replaced with FBS-free medium containing 5% fatty acid-free bovine serum albumin (BSA, Sigma) overnight, and then incubated with PA in the presence of serum vehicle or serum-containing TDQ for 24 h. PA was prepared according to a previously described procedure [21, 22]. PA was dissolved in 0.1 mol/L NaOH to a concentration of 100 mmol/L by heating at 70°C in a shaking water bath, and the solution was then diluted with 5% BSA DMEM at a stock solution of 2.5 mmol/L at 55°C in a shaking water bath. After filtration (0.22 *μ*m pore size membrane filter), this solution was stored at -20°C for further use. Stored stock solution was heated for 15 min at 55°C and then cooled to room temperature before use.

The cells in the normal group (Nor) were grown in 5% BSA DMEM and 10% serum vehicle, the cells in the model group (Mod) were grown in PA and 10% serum vehicle, and the cells in 5% or 10% TDQ were grown in PA and 5% or 10% drug-containing serum vehicle. Then, the supernatant media were harvested to test glucose consumption according to the manufacturer's instructions (glucose oxidase method, BioSino Bio-Technology & Science Inc., Beijing, China). In brief, the amounts of glucose consumption were calculated by subtracting the glucose concentration of TDQ-treated cells from that of the medium without cell conditioning. Glucose concentration was normalized to the protein levels and presented as mmol glucose/g protein. And for the intracellular triglyceride (TG) test, cells were washed three times with PBS and lysed with 100 *μ*L ethanol : acetone (1 : 1) mixture for 30 minutes at room temperature with constant agitation, centrifuged at 10000g for 5 min. The supernatant was collected for the TG content test and normalized to cellular protein levels. Cells were also harvested according to the procedure for RT-PCR and western blotting analysis. Three independent tests for every experiment were performed each time in triplicates.

### 2.4. Measurement of FBG and Oral Glucose Tolerance Tests (OGTT)

Before and after 5 weeks of intragastric administration, rats were fasted for 12 h with free access to water; tail venous blood samples were collected for the measurement of FBG. Thereafter, the 12 h-fasted rats in all groups were intragastrically administered with 50% glucose (2.0 g/kg) [23]; blood glucose was determined from the tail vein at regular intervals (30, 60, and 120 min). The OGTT curve was drawn, and the area under the curve (OGTT-AUC) was calculated.

### 2.5. Sample Collection and Biochemical Index

The rats were sacrificed after 5 weeks of treatment. Blood samples were obtained and then centrifuged at 3000 r/min for 15 min at 4°C; the serum was stored at -20°C until further analyses. Fresh liver samples were immediately collected using ice cubes, some livers were fixed in 10% formaldehyde, and some were stored at -80°C for further analyses.

Concentrations of fasting serum insulin (FINS) in the serum were determined with radioimmunoassay by using kits from Beijing Sino-UK institute of Biological Technology (Beijing, China). The homeostasis model of assessment for insulin resistance was computed as follows: HOMA-IR = [fasting glucose (mmol/L) × fasting insulin (*μ*U/mL)]/22.5. The homeostasis model of assessment for insulin sensitivity was computed as follows: HOMA-IS = Ln [1/(FBG × FINS)] [24, 25].

Leptin and adiponectin levels in the serum were measured by using an ELISA kit according to their manufacturer's instructions (Invitrogen Corporation, Carlsbad, CA, USA). Concentrations of TG and total cholesterol (TC) in the serum and TG in the liver were determined using colorimetric kits (BioSino Bio-Technology & Science Inc. and Beijing Sino-UK Institute of Biological Technology) with a spectrophotometer, according to the manufacturer's instructions.

### 2.6. Hepatic Histology Analysis

For haematoxylin-eosin staining, the liver was fixed in 10% formaldehyde, after being fixed for 24 h and dehydrated; the specimens of the liver were embedded in paraffin, sliced into sections of 5 *μ*m thickness, and stained with haematoxylin-eosin. Steatosis was graded as follows: 0, 0% to 5% of the hepatocytes in the section are steatotic; 1, greater than 5% to 33% of hepatocytes are steatotic; 2, greater than 33% to 66% of hepatocytes are steatotic; and 3, greater than 66% of hepatocytes are steatotic [26].

### 2.7. Western Blotting

Liver tissues and HepG2 cells were prepared for protein extraction. Total protein was obtained using RIPA buffer with PMSF (Beyotime) for 30 min followed by centrifugation at 12000 rpm for 10 min at 4°C, while the nuclear protein was acquired by using a Nuclear Protein Extraction kit (Beyotime). The concentrations were determined by using a BCA protein assay. Total protein was subjected to SDS-PAGE gel electrophoresis and electroblotted onto PVDF membranes (Millipore, USA). The membranes were blocked for 1 h in 5% BSA and subsequently incubated with the following primary antibodies at 4°C overnight: primary antibodies against GADPH (rabbit monoclonal antibody 1 : 1000, Santa Cruz), PGC-1*α* (rabbit monoclonal antibody 1 : 1000, Abcam), PPAR*γ* (mouse monoclonal antibody 1 : 1000, Santa Cruz), and DGAT2 (mouse monoclonal antibody 1 : 500, Santa Cruz). The membrane was then incubated with the secondary antibody (1 : 5000; anti-rabbit or anti-mouse IgG conjugated with horseradish peroxidase, Solarbio Life Science, Beijing, China). And then, the conjugates were visualized with an ECL system. The relative density of each protein band was analyzed by an imaging densitometer (Cuene Genins). Densitometry values were normalized with GADPH.

### 2.8. RT-PCR Analysis

Total RNA was isolated from the liver samples and HepG2 cells by using an RNA simple total RNA kit (Tiangen, Beijing) and reverse transcribed into cDNA according to the manufacturer's protocol (Tiangen, Beijing). The cDNA was then amplified by PCR using the sense primer:

1. GAPDH: 5′-ATGCTGGCGCTGAGTACGTC-3′ and 5′-GGGCAGAGATGATGACCCTT-3′
2. DGAT2: 5′-TGGGGGCTGGTGCCCTACTC-3′ and 5′-AATTGGCCCCGAAGGCTGGC-3′
3. PPAR*γ*: 5′-TCTCTCCGTAATGGAAGACC-3′ and 5′-GCATTATGAGACATCCCCAC-3′

The 2^-△△Ct^ method was used to calculate relative levels of mRNA expression after normalization with those of GAPDH as a housekeeping gene.

### 2.9. Statistical Analyses

Statistical analyses were performed using SPSS version 23.0 (IBM Inc.). For the comparison of multiple groups, parametric data were compared by one-way ANOVA with Bonferroni's post hoc test, and nonparametric data were compared by a Kruskal-Wallis 1-way ANOVA test. *P* < 0.05 was considered statistically significant. Values were presented as the means ± SD.

## 3. Results

### 3.1. TDQ Improve Glucose Tolerance and Insulin Resistance in Type 2 Diabetes Rats

**Figure 1:**
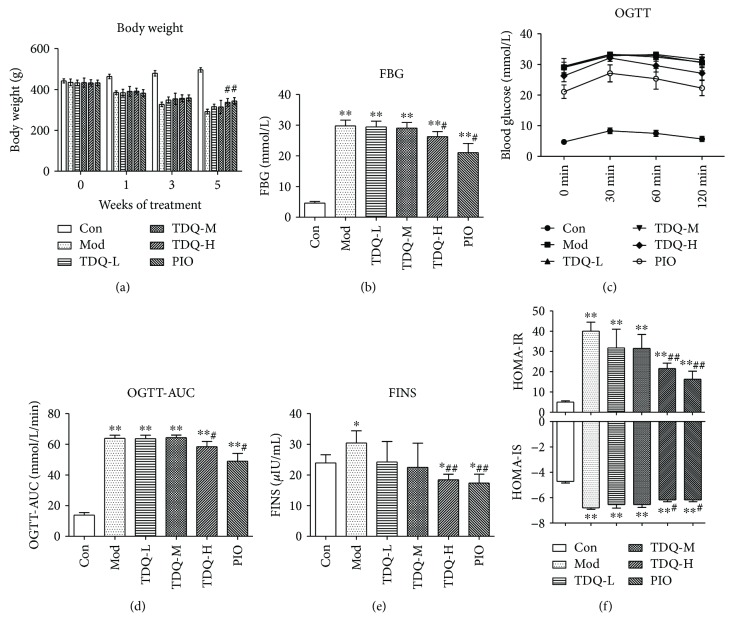
TDQ mitigated the insulin resistance of type 2 diabetic rats. (a) Body weight over time in each groups. (b–d) Fasting blood glucose (FBG) and oral glucose tolerance tests (OGTT) and area under the curve (OGTT-AUC) were calculated in rats at the end of the 5-week experimental protocol. (e, f) Circulating insulin (FINS), calculated HOMA-IR, and HOMA-IS in each group. Results are shown as the mean ± SD. ^∗^*P* < 0.05 and ^∗∗^*P* < 0.01 vs. Con; ^#^*P* < 0.05 and ^##^*P* < 0.01 vs Mod. Con: control group, Mod: model group, TDQ: Tangduqing granules, PIO: pioglitazone group.

Compared to that of the control (Con) group, the body weight of the model (Mod) group reduced dramatically. As illustrated in Figure 1(a), in comparison with the Mod group, supplemented with high dose of Tangduqing granules (TDQ-H) or pioglitazone (PIO), body weight obviously increased after 5 weeks of intervention. FBG levels were determined before and after treatment (Figure 1(b)), after five weeks of treatment. FBG levels of the TDQ-H and PIO groups were descended. The OGTT levels and the OGTT area under the curve (OGTT-AUC) are shown in Figures 1(c) and 1(d). The blood glucose of each group reached the peak in 30 min, and thereafter, the level showed a downward trend in all groups. Diabetic rats showed impaired glucose tolerance compared with the Con group. The values of OGTT-AUC in the TDQ-H and PIO groups were significant lower than that of the Mod group, which suggested that TDQ-H and PIO notably improved glucose tolerance. Type 2 diabetic rat insulin resistance was accompanied by hyperinsulinaemia, Figure 1(e) shows that the FINS level in the Mod group (30.44 ± 3.95 *μ*IU/mL) was significantly higher than that in the Con group (23.94 ± 2.63 *μ*IU/mL) (*P* < 0.05). After five weeks of treatment, compared with that of the Mod group, the FINS of all treatment groups was reduced. It is interesting that the FINS levels in the TDQ-H and PIO groups further decreased compared to that in the Con group. We further assess insulin resistance and insulin sensitivity by homeostasis model assessment.  Figure 1(f) shows that in the Mod group, the HOMA-IR value was dramatically higher and the HOMA-IS value was significantly lower than that in the Con group. The HOMA-IR and the HOMA-IS in the TDQ-H and PIO groups were markedly improved compared with those in the Mod group. These findings indicate that the TDQ-H can effectively decrease blood glucose and ameliorate insulin resistance in type 2 diabetes rats.

### 3.2. TDQ Attenuate Lipid Metabolic Disturbance in Type 2 Diabetes Rats

**Figure 2:**
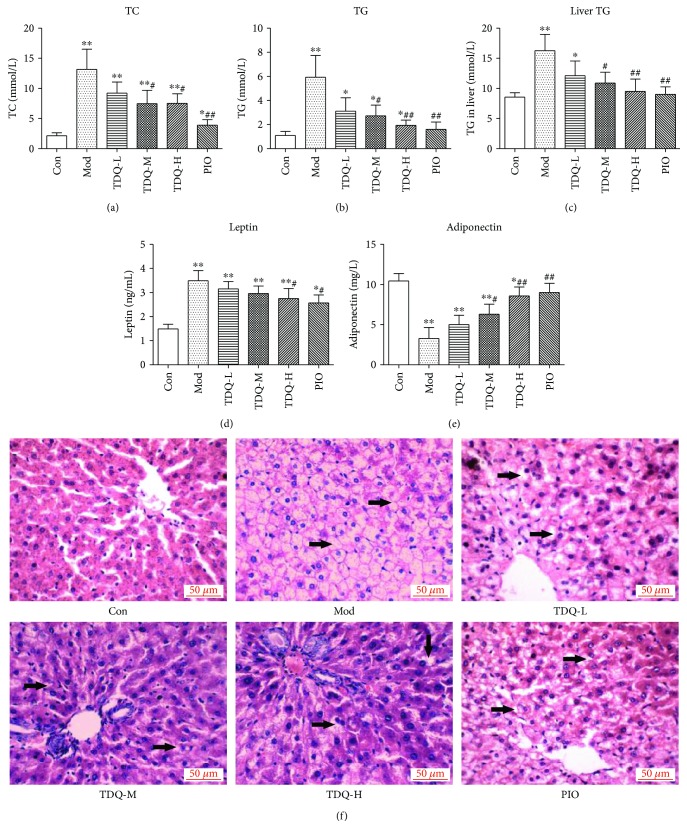
TDQ impaired lipid metabolism disorder in diabetic rats. (a–c) Biochemistry determination in serum and liver tissue of total cholesterol (TC) and triglycerides (TG). (d, e) Leptin and adiponectin levels in serum. (f) Histological analysis of hepatic tissues in each group. Steatosis and vacuolization (arrows). Conventional H&E staining was performed (magnification: 400x). Data are presented as the mean ± SD. ^∗^*P* < 0.05 and ^∗∗^*P* < 0.01 vs. Con; ^#^*P* < 0.05 and ^##^*P* < 0.01 vs. Mod.

To further verify that TDQ could moderate many of the symptoms present in type 2 diabetes, serum and hepatic lipid levels were assayed (Figures 2(a)–2(c)). The serum TC and TG levels of the model rats significantly increased (*P* < 0.01), as well as the hepatic TG level when compared to the control rats. After five weeks of treatment, the rats in the TDQ and PIO groups exhibited significant reduction in TC and TG levels. Leptin and adiponectin levels (Figures 2(d) and 2(e)) in the serum were measured, which affect fat and carbohydrate metabolism and insulin sensitivity. The leptin level was obviously increased, whereas the adiponectin level was decreased markedly in the Mod group when compared to the Con group. Administration with TDQ or PIO partly restored the leptin and adiponectin concentration, and the levels in the TDQ-H and PIO groups were more pronounced than others.

Some studies have suggested that excessive cholesterol and triglyceride accumulation results in the progression of a fatty liver [13, 27]. Next, liver histology was detected by H&E staining (Figure 2(f)). In the H&E sections, the diabetes rats developed obvious steatosis and vacuolization (arrows) accompanied by lobular inflammation and hepatocyte balloon-like changes compared to the Con group, whereas TDQ-H treatment notably ameliorated fat vacuole degeneration of the liver compared to the Mod group. Liver sections were blindly evaluated for steatosis scores; these data demonstrate that all normal mice were negative (grade 0), and 60% of the Mod group had grade 3 lesions, with the 30% having grade 2 changes; while drug treatment decreased lesion grade, TDQ-H and PIO-treated ameliorated the most (Table 1). Taken together, these results indicate that TDQ can affect lipid metabolic parameters and effectively ameliorate lipid metabolism and hepatic steatosis in type 2 diabetes rats.

### 3.3. PPAR*γ* and DGAT2 Play Potential Roles in STZ-Mediated Metabolic Abnormalities

**Figure 3:**
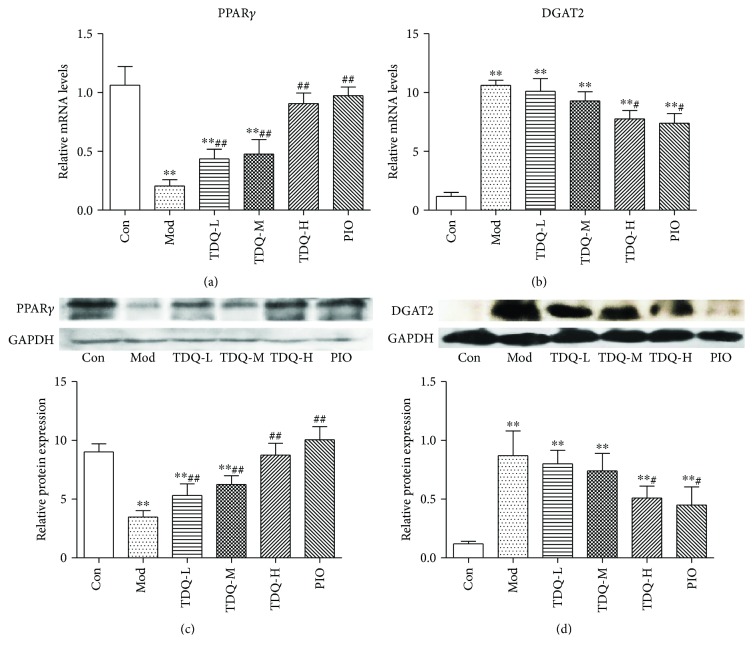
PPAR*γ* and DGAT2 play crucial roles in high-fat diet and STZ-mediated insulin resistance and abnormal lipid metabolism diabetic model. (a, b) qRT-PCR analysis of mRNA levels of PPAR*γ* and DGAT2. (c, d) Western blotting showing PPAR*γ* and DGAT2 protein expression in liver and quantitative analysis of protein expression relative to GAPDH. Data are presented as the mean ± SD. ^∗^*P* < 0.05 and ^∗∗^*P* < 0.01 vs. Con; ^#^*P* < 0.05 and ^##^*P* < 0.01 vs. Mod.

To explore the underlying mechanism behind TDQ's intervention in glucose and lipid metabolism disorder, we analyzed the gene and protein changes of PPAR*γ* and DGAT2 expression in the livers (Figure 3). In the model group, the mRNA and protein expression of PPAR*γ* was significantly decreased, and the mRNA and protein expression of DGAT2 was significantly elevated. However, TDQ-H and PIO treatment obviously facilitated PPAR*γ* expression and downregulated DGAT2 expression compared with the Mod group. These observations suggest that TDQ modulate STZ-mediated metabolic abnormalities by inducing PPAR*γ* expression and dissociating subsequent effects on DGAT2.

### 3.4. TDQ Ameliorate Glucose Consumption and Intracellular Lipid Deposition in PA-Treated HepG2 Cells

**Figure 4:**
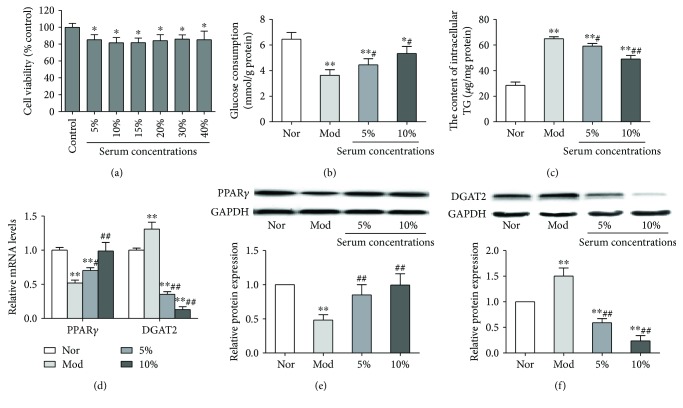
Effects of TDQ on palmitate-induced hepatocellular model of insulin resistance. (a) Cell viability of HepG2 cells treated with different concentrations of TDQ-containing serum. (b, c) Glucose consumption and intracellular TG were analyzed in TDQ-treated HepG2 cells. (d, f) mRNA and protein levels of PPAR*γ* and DGAT2 in palmitate-induced insulin resistance in HepG2 cells. Data are presented as the mean ± SD. ^∗^*P* < 0.05 and ^∗∗^*P* < 0.01 vs. Nor; ^#^*P* < 0.05 and ^##^*P* < 0.01 vs. Mod.

Based on the results of TDQ on diabetes rats, the effect of TDQ on a hepatocellular model of insulin resistance and lipid metabolic abnormalities was evaluated. First, the cell viability of HepG2 cells treated with TDQ-containing serum was examined (Figure 4(a)). The cell viability in different TDQ-containing serum groups were significantly reduced as compared to that in the Nor group, and 5% and 10% serum concentrations were selected for a further study. We observed significant alteration of glucose consumption in HepG2 cells after PA exposure, whereas supplementation with drug-containing serum significantly increased glucose consumption (Figure 4(b)). The intracellular TG in the PA-treated group was markedly increased, while drug-containing serum supplementation could partly alleviate that (Figure 4(c)). Palmitate treatment also caused downregulation of PPAR*γ* and upregulation of DGAT2 in mRNA and protein levels, but supplementation with TDQ-containing serum could reverse these alterations (Figures 4(d) and 4(e)). These findings again suggest that TDQ modulate glucose and lipid metabolic abnormalities through synergistic interaction of PPAR*γ* and DGAT2.

## 4. Discussion

Diabetes belongs to “Xiaoke disease” of the category in Chinese medicine, and its pathogenesis is “Yin deficiency and dryness heat”; one symptom is characterized by spleen deficiency in scattering and removing turbidites [28, 29]. Tangduqing granules (TDQ), a Chinese medicinal formula, were composed of several Chinese herbs, such as Coptis chinensis Franch., *Rheum palmatum*, Scutellaria baicalensis Georgi, and Paeonia lactiflora Pall, with the effects of clearing heat and dampness, purging fire and detoxification and nourishing Yin in traditional Chinese medicine's theoretical system. In contemporary research, some drugs have been reported that have the effect of lowering blood glucose, blood lipids, and lipid metabolism and improving insulin resistance [30, 31]. Evidence from our previous experiment has demonstrated that TDQ based on removing turbidites and detoxification methods work effectively against lipid and glucose metabolism disorder in clinical setting [18]. The present study discloses that TDQ play a crucial role in treating insulin resistance of type 2 diabetes. It demonstrates that TDQ treatment, associated with increased hepatic PPAR*γ* and reduced DGAT2 expression, targets multiple risk factors of the type 2 diabetes; additionally, it is a glucose- and lipid-lowering agent with a strong ability to treat diabetic complications.

Type 2 diabetes is owing to IR, characterized by a deficient action of the peripheral tissues, including skeletal muscles, adipose tissues, and liver, in response to insulin [32, 33]. In the present study, we successfully established the HFD/STZ rat model mimicking human type 2 diabetes with impaired insulin secretion and insulin resistance. In agreement with these, it has been shown that diabetic rats had significantly decreased body weight but increased fasting blood glucose, oral glucose tolerance, fasting serum insulin, and insulin resistance index as well as lipid levels. Interestingly, the results demonstrate that TDQ effectively ameliorate hyperlipidemia, hyperglycaemia, and insulin resistance of diabetic rats after 5 weeks of treatment.

Leptin and adiponectin are considered to be the most important adipokines with potential metabolic effects. Leptin, a peptide hormone secreted by adipose tissue, has been shown to be an effective factor in food intake and energy consumption that regulates central body fat distribution and body weight [34]. Our present study showed that the leptin level was significantly increased in diabetic rats and body weight was obviously lost, and these alterations were partly reversed after treatment with TDQ. Adiponectin is an adipocyte-derived plasma protein that participates in the regulation of carbohydrate and lipid metabolism. It reduces TG content in the skeletal and cardiac muscle as well as in the liver and inhibits glucose production by the liver; consequently decreasing blood glucose levels [35]. Our experiments reported that adiponectin obviously decreased, and TG content and fasting blood glucose levels significantly increased in the model group, while TDQ treatment reversed these alterations. Moreover, leptin and adiponectin also modulate insulin activity and sensitivity. It has been reported that there was a direct correlation between circulating leptin levels and insulin resistance markers, while a negative correlation of adiponectin was found with insulin resistance markers [36, 37]. These findings support the alterations in fasting serum insulin and insulin resistance index (HOMA-IR and HOMA-IS) of type 2 diabetic rats. Histopathology confirmed the ability of TDQ treatment to decrease hepatic steatosis area and to normalize tissue structure.

Multiple mechanisms are involved in IR of type 2 diabetes. We suggest that this ability of TDQ to provide multifaceted actions in the IR of type 2 diabetes can be attributed to its biological actions associated with PPAR*γ* activation and DGAT2 inhibition. PPAR*γ* and DGAT2 have been shown to play key roles in maintaining glucose and lipid homeostasis by acting as insulin sensitizers and lipid-lowering agents, respectively [10, 12]. Expression of DGAT2 gene is involved in TG synthesis [38]; meanwhile, it has been reported that PPAR*γ* might be involved in HFD-induced liver steatosis. Mountains of evidence have shown that PPAR*γ* was normally expressed at low levels in the liver and increased in the fatty liver [39, 40]; however, our data demonstrated that PPAR*γ* was decreased in the liver of type 2 diabetic rats which is opposite with these reports. We also found opposite situations that PPAR*γ* was expressed lower in the liver of a rat model of type 2 diabetes or in white adipose tissue of high-fat diet-induced obese mice [41, 42]. So we supposed that the expression of PPAR*γ* may be a dynamic change process in a different phase of a different model, and the dynamic changes are worthy of further research.

Our findings collectively demonstrate that TDQ, as a Chinese medicinal formula, prevent the development of the metabolic syndrome and reduce the severity of type 2 diabetes. It can act on multiple cardinal metabolic events, may enhance its beneficial effects, and may reduce unwanted adverse effects, which could be helpful in finding a new strategy for attenuation of IR and metabolic abnormality. However, we only found some clues that TDQ could improve lipid and glucose metabolism disorder and insulin resistance in diabetic rats and HepG2 cells; there is still a lot of work to do in the future study; for example, we need to explore the effect of TDQ on primary hepatocytes and to disclose which components play on metabolic events and mechanism.

## 5. Conclusion

In summary, the present study demonstrated that the widely used traditional Chinese medicinal formula “Tangduqing granules” was capable of reversing insulin resistance and lipid metabolism disorder in diabetic rats, at least partly through coordinated regulation of PPAR*γ* and DGAT2 signal molecules. These findings indicated that Tangduqing granules may act as a potential candidate drug in treating insulin resistance and abnormal lipid metabolism of type 2 diabetes mellitus through a multitarget way, which elucidated the scientific basis of the traditional Chinese medicine formula and illustrated the rational guidance for clinical application.

## Figures and Tables

**Table 1:** Hepatic steatosis scores.

	No. of rats			
	0	1	2	3
Con	10	0	0	0
Mod	0	1	3	6
TDQ-L	0	2	4	4
TDQ-M	0	2	5	3
TDQ-H	0	5	3	2
PIO	0	6	3	1

## Data Availability

The data used to support the findings of this study are available from the corresponding author upon request.
